# The effectiveness of dry needling at myofascial trigger points for knee disorders: A quantitative synthesis of randomized controlled trials

**DOI:** 10.1371/journal.pone.0346129

**Published:** 2026-04-10

**Authors:** Xin Hu, Ting Lei, Zheng Liu, Zhenchao Xu, Guanghui Zhu

**Affiliations:** 1 Department of Orthopedics, The Affiliated Children’s Hospital of Xiangya School of Medicine, Central South University (Hunan Children’s Hospital), Hunan Provincial Key Laboratory of Pediatric Orthopedics, Changsha, Hunan, PR China; 2 The School of Pediatrics, University of South China, Changsha, Hunan, PR China; 3 Furong Laboratory, Changsha, PR China; 4 MOE Key Lab of Rare Pediatric Diseases, University of South China, Changsha, Hunan, PR China; University College London, UNITED KINGDOM OF GREAT BRITAIN AND NORTHERN IRELAND

## Abstract

**Purpose:**

Dry needling (DN) targeting myofascial trigger points (MTrPs) has been proposed as a treatment for knee disorders, including knee osteoarthritis (KOA) and patellofemoral pain syndrome (PFPS). This meta-analysis evaluated the effectiveness of DN in improving pain and function in patients with knee disorders.

**Methods:**

This meta-analysis was conducted in accordance with PRISMA 2020 guidelines and was prospectively registered in PROSPERO (CRD420261294603). Systematic searches were performed in PubMed, Embase, Web of Science, Cochrane CENTRAL, CNKI, Wanfang, and VIP databases from inception to December 2025 for randomized controlled trials (RCTs) comparing DN targeting MTrPs with sham DN, no intervention, or other active treatments for knee disorders. Primary outcomes were pain intensity measured by the Visual Analog Scale (VAS) and Numeric Pain Rating Scale (NPRS). Secondary outcomes included functional status assessed by the WOMAC functional subscale and the Kujala Patellofemoral Score. Weighted mean differences (WMDs) were calculated using random-effects models. Risk of bias was assessed using the Cochrane Risk of Bias 2 (RoB 2) tool, and certainty of evidence was evaluated using the GRADE framework.

**Results:**

Twenty RCTs (n = 1,234; mean age range: 22–69 years) met the inclusion criteria. Compared with controls, DN significantly reduced knee pain across all pain measures: NPRS (WMD = –1.00, 95% CI: –1.25 to –0.76; *I²* = 0.0%), VAS (WMD = –1.19, 95% CI: –1.73 to –0.66; *I²* = 80.4%), and WOMAC Pain subscale (WMD = –1.76, 95% CI: –2.57 to –0.95; *I²* = 67.6%), with an overall pooled pain reduction of WMD = –1.25 (95% CI: –1.58 to –0.92; *I²* = 74.7%). DN also significantly improved knee function as measured by the WOMAC functional subscale (WMD = –6.59, 95% CI: –8.88 to –4.29; *I²* = 61.6%) and the Kujala Patellofemoral Score (WMD = 6.39, 95% CI: 4.64 to 8.14; *I²* = 30.1%). Pre-specified sensitivity analyses using standardized mean differences confirmed the robustness of these findings. The overall risk of bias was moderate, with concerns primarily related to inadequate blinding of participants and outcome assessors. The GRADE certainty of evidence was rated as moderate for all primary outcomes.

**Conclusion:**

DN targeting MTrPs provides significant short-term pain relief and functional improvement in KOA and PFPS, with pain reductions approaching clinically important thresholds. However, substantial heterogeneity, blinding limitations, and short follow-up necessitate cautious interpretation, and high-quality long-term RCTs are needed.

## Introduction

Knee disorders, particularly knee osteoarthritis (KOA) and patellofemoral pain syndrome (PFPS), are among the most prevalent musculoskeletal conditions worldwide, substantially impairing mobility and quality of life [1]. KOA is a degenerative joint disease characterized by progressive cartilage loss, resulting in pain, stiffness, and functional limitations, especially in older adults [2]. In contrast, PFPS is more common in younger, physically active populations, presenting as anterior knee pain often associated with overuse or suboptimal biomechanics during activity [3]. Epidemiological estimates suggest that approximately 10% of adults aged ≥60 years have symptomatic KOA [4], whereas PFPS affects 25%–40% of active adolescents and young adults [5]. Risk factors for both conditions include age, obesity, and repetitive knee-loading occupations (e.g., construction, agriculture). The growing prevalence of knee disorders contributes to rising healthcare expenditure, long-term disability, and productivity loss, imposing substantial socioeconomic burdens globally.

Myofascial trigger points (MTrPs) are localized, hyperirritable spots within taut skeletal muscle fibers or fascia, often associated with characteristic pain referral patterns [6]. MTrPs have been identified in muscles surrounding the knee in both KOA and PFPS. In KOA, MTrP prevalence ranges from approximately 11% to 50% across several lower-limb muscles, with greater latent MTrP burden moderately associated with disability; commonly affected muscles include the quadriceps, hamstrings, and gastrocnemius [7]. In PFPS, higher MTrP prevalence has been documented in hip/thigh and lumbo-pelvic muscles compared with controls, suggesting a broader myofascial contribution to anterior knee pain [8]. In PFPS, MTrPs in the quadriceps and adjacent musculature may contribute to pain and neuromuscular dysfunction; however, the role of patellar tracking remains debated, with variable measurement reliability and with variable measurement reliability and no established causal links to symptoms to symptoms. In KOA, MTrPs in the hamstrings and calf muscles may exacerbate stiffness and gait alterations, further limiting mobility [9].

Dry needling (DN) is a minimally invasive intervention involving the insertion of solid filiform needles into MTrPs for therapeutic purposes [10]. Unlike acupuncture, which is based on meridian theory, DN is grounded in musculoskeletal anatomy and aims to elicit local twitch responses to inactivate trigger points [11]. This physiological response is believed to reduce muscle tension, decrease nociceptive input, and improve function. DN has been applied to periarticular muscles in both PFPS and KOA, with studies reporting reductions in pain intensity and improvements in function [12,13]. Despite these encouraging findings, current evidence is limited by small sample sizes, heterogeneous protocols, and variable follow-up durations.

Notwithstanding their clinical relevance, both the MTrP concept and DN’s mechanisms remain debated. Quintner et al. questioned the validity of MTrPs as discrete pathological entities, while Braithwaite et al. showed that inadequate blinding in DN trials can exaggerate perceived analgesic effects [14,15]. These considerations highlight the need for cautious interpretation and high-quality randomized controlled trials (RCTs) with robust methodology.

Previous systematic reviews have investigated DN for musculoskeletal pain, but few have focused specifically on KOA and PFPS, and most lacked comprehensive quantitative synthesis stratified by diagnosis or outcome domain. Many excluded recent RCTs or failed to evaluate both pain and function simultaneously. Therefore, the present meta-analysis synthesizes data from 20 RCTs, assessing DN’s effectiveness in KOA and PFPS, with updated risk-of-bias assessments and evidence grading, to provide more reliable clinical guidance.

## Methods

### Study selection criteria

The risk of bias in included RCTs was assessed using the Cochrane Risk of Bias 2 (RoB 2) Tool [16], and reporting followed the PRISMA 2020 Guidelines [17]. To enhance transparency and methodological rigor, this systematic review and meta-analysis was registered in the International Prospective Register of Systematic Reviews (PROSPERO, Registration number: CRD420261294603). A PRISMA 2020 checklist has been provided as supplementary material (Supplementary File 2).

Eligible studies were identified based on a structured PICO framework. The Population (P) comprised adults aged 18 years or older diagnosed with knee disorders associated with MTrPs, including knee osteoarthritis (KOA), patellofemoral pain syndrome (PFPS), or other musculoskeletal knee conditions with documented MTrPs in periarticular muscles such as the quadriceps, hamstrings, gastrocnemius, or gluteus medius. The Intervention (I) was DN specifically targeting MTrPs in muscles surrounding the knee, administered by trained practitioners using solid filiform needles. The Comparison (C) included sham DN (non-penetrating or superficial needling), no intervention or waitlist control, standard conservative care (such as exercise therapy, manual therapy, or pharmacotherapy), or other active interventions (such as corticosteroid injection or ultrasound therapy). The Outcomes (O) of interest were pain intensity measured by the Visual Analog Scale (VAS), Numeric Pain Rating Scale (NPRS), or WOMAC Pain subscale, and functional status assessed using the Western Ontario and McMaster Universities Osteoarthritis Index (WOMAC) Function subscale, Kujala Patellofemoral Score, Knee Society Score (KSS), or Knee injury and Osteoarthritis Outcome Score (KOOS). Secondary outcomes included pressure pain threshold (PPT), quality of life measures, and adverse events when reported. Only RCTs with parallel-group or crossover designs were included. Studies were required to report sufficient statistical data (mean, standard deviation, and sample size) for at least one primary outcome at any follow-up time point. No language or publication date restrictions were applied during the search phase.

Studies were excluded if they were non-randomized, lacked a control group, applied DN without targeting MTrPs in the knee region, or failed to report valid pain or functional outcome measures. Trials with unclear diagnostic criteria for knee disorders or insufficient data for quantitative synthesis were also excluded.

### Data sources and search strategy

A comprehensive literature search was conducted from database inception to December 31, 2025, to identify all relevant RCTs on DN for knee disorders. The following databases were searched: PubMed, Embase, Web of Science, Cochrane CENTRAL, CNKI, Wanfang, and VIP databases from inception to December 2025, without language restrictions. Search strategies combined controlled vocabulary (e.g., MeSH terms) and free-text keywords related to DN, MTrPs, knee pain, knee osteoarthritis, patellofemoral pain syndrome, and RCTs. The complete search strings, including Boolean operators, filters, and applied limits for each database, are provided in Supplementary File 1. Additionally, the reference lists of eligible articles and relevant reviews were screened to identify any further eligible studies.

### Data extraction

Two independent reviewers (X.H. and T.L.) conducted the search, screened titles and abstracts, and extracted data. Disagreements were resolved by consensus or consultation with a third reviewer (Z.L.). Extracted data included: author(s), publication year, sample size, patient characteristics (age, diagnosis), DN protocol (technique, frequency, duration, adjunct therapies), and outcome measures. Pain intensity measured by NPRS and VAS was designated as the co-primary pain outcome, given their validated psychometric properties and comparable 0–10 numerical scales. The WOMAC Pain subscale was treated as a secondary pain outcome, as it is scored on a different scale (0–20 or 0–100) and reflects pain within the broader context of osteoarthritis symptomatology.

### Assessment of risk of bias

The methodological quality of all included RCTs was assessed using the RoB 2 tool, which evaluates potential biases across five key domains: (1) randomization process (including random sequence generation and allocation concealment), (2) deviations from intended interventions (blinding of participants and personnel), (3) missing outcome data, (4) measurement of the outcome (including blinding of outcome assessors, particularly for subjective measures such as VAS and WOMAC), and (5) selection of the reported result (selective outcome reporting). For selective reporting, we compared the outcomes presented in the final publication against those specified in trial registries or the study’s stated objectives. When reporting for a given domain was insufficient or unclear, the risk of bias was rated as “unclear.” Publication bias was evaluated by visually inspecting funnel plots for asymmetry, which may indicate selective publication of positive findings; where applicable, Egger’s regression test was performed to provide a quantitative assessment.

### Statistical analysis

Statistical analyses were performed using RevMan 5.4 and Stata 17.0 (StataCorp LLC). Weighted mean differences (WMDs) with 95% confidence intervals (CIs) were used as the primary effect measure for all outcomes, as studies within each comparison used the same measurement scale (NPRS and VAS: 0–10; WOMAC subscales: 0–100; Kujala: 0–100). WMDs were selected because they preserve the original measurement unit and facilitate direct clinical interpretation. Pre-specified sensitivity analyses were conducted using standardized mean differences (SMDs, Hedges’ g) to confirm the robustness of findings across outcomes and to enable cross-scale comparison. SMDs were interpreted using Cohen’s benchmarks: small (0.2–0.5), moderate (0.5–0.8), and large (≥0.8). All analyses employed random-effects models (DerSimonian–Laird method). Heterogeneity was quantified using the *I²* statistic and interpreted as low (<25%), moderate (25–75%), or high (>75%). Subgroup analyses were pre-specified by disease type (KOA vs. PFPS) and follow-up duration (<4 weeks vs. ≥ 4 weeks). Leave-one-out sensitivity analyses were performed to evaluate the stability of pooled estimates.

## Results

### Study selection and characteristics

**Fig 1:**
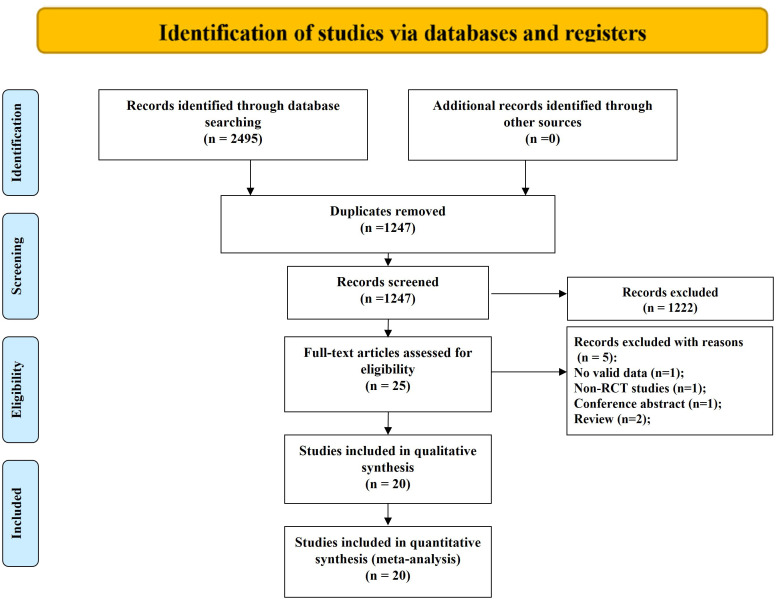
Study identification and selection. Original figure created by author; licensed under CC BY 4.0.

The study selection process is summarized in Fig 1. A total of 2,495 records were retrieved from database searches. After removing duplicates, 1,247 titles and abstracts were screened for relevance. Twenty-five full-text articles were assessed for eligibility, of which five were excluded for the following reasons: invalid or incomplete data (n = 1), non-randomized design (n = 1), conference abstract only (n = 1), and review articles (n = 2). Details of excluded studies are provided in S6 Table. Ultimately, 20 RCTs (n = 1,234) met the inclusion criteria and were included in both qualitative synthesis and quantitative meta-analysis [18–37].

Baseline demographic and clinical characteristics of the included study populations are summarized in Tables 1 and S1. Across the 20 RCTs, participants represented a broad spectrum of age, disease severity, and activity levels. Trials on KOA predominantly enrolled older adults, with mean ages ranging from 56 to 75 years, and in studies reporting radiographic classification, most patients had Kellgren–Lawrence grade II–III disease [22,32,33]. In contrast, trials on PFPS generally involved younger and physically active individuals, with mean ages between 20 and 29 years [18,19]. A smaller number of studies investigated myofascial pain syndrome affecting the knee region. Symptom duration varied widely: some PFPS and MPS studies reported subacute presentations (>2 weeks to 6 weeks; [27,28], whereas several KOA studies included participants with long-standing chronic pain exceeding one year [22]. Activity levels also differed by diagnosis, with PFPS cohorts more likely to engage in regular sports or high-load knee activities, while KOA cohorts were often sedentary or engaged in low-intensity daily activities. Prior and concurrent treatments varied: some studies enrolled participants naïve to structured interventions [19,30], others excluded those with recent intra-articular injections or surgeries, and a few specifically targeted post-operative populations [24]. Geographically, the studies were conducted across Iran, USA, Spain, China, Belgium, and Italy, ensuring diversity in clinical settings and healthcare contexts.

**Table 1:** Characteristics of all studies.

No.	Author (year)	Study design, Region	Sample of study	Age (Years) (T/C)	Enrolled patient	Pain duration	Intervention	Follow-up time	Outcome index	Funding Reported (Yes/No)
1	Eleuterio A. Sánchez-Romero (2018)	RCT, Spain	20	71.89 ± 4.80/ 70.89 ± 3.21	Knee osteoarthritis	> 30 days	DN + Exercise vs. Exercise	12 weeks	NPRS, WOMAC_Pain, WOMAC_Functional	No
2	Eleuterio A. Sánchez-Romero (2020)	RCT, Spain	62	72.97 ± 6.29/ 71.65 ± 5.00	Knee osteoarthritis	NR	DN + Exercise vs. Exercise	48 weeks	WOMAC_Pain, WOMAC_Functional, WOMAC Stiffness	Yes
3	Fereshteh Karamiani (2022)	RCT, Iran	29	24.4 ± 3.94/ 27.64 ± 7.13	Patellofemoral pain syndrome	>4 weeks	Physiotherapy + DN (GM MTrP) vs. Physiotherapy	1 week	VAS, Kujala score	Yes
4	GEMMA V. ESPÍ-LÓPEZ (2017)	RCT, USA	60	29.2 ± 10.5/ 29.7 ± 9.5	Patellofemoral pain syndrome	≥ 6 months	Manual therapy + Exercise + DN vs. Manual therapy + Exercise	12 weeks	NPRS, KSS function subscale	No
5	Hanieh Zarei (2020)	RCT, Iran	40	22.25 ± 3.25/ 25.65 ± 8.49	Patellofemoral pain syndrome	NR	DN + Exercise vs. Exercise	6 weeks	NPRS, Kujala score, Step-down test, PPTQL, PPTGM	No
6	James Dunning (2018)	RCT, Italy	242	57.1 ± 13.2/ 58.1 ± 13.1	Knee osteoarthritis	4.5 ± 4.7 years	Manual therapy + Exercise + electrical DN vs. Manual therapy + Exercise	12 weeks	WOMAC_Pain, WOMAC_Functional, WOMAC Stiffness	No
7	John S. Mason (2016)	RCT, USA	39	20.3 ± 1.08/ 20.16 ± 2.12	Atraumatic knee pain	>2 weeks	DN (hamstring MTrPs) vs. Sham DN	1 week	VAS, PPT	No
8	Jorge Velázquez Saornil (2017)	RCT, Spain	42	31.4 ± 8.3/ 34.4 ± 8.6	After ACL arthroscopic reconstruction surgery	NR	Rh + DN (vastus medialis MTrP) vs. Rh	5 weeks	VAS, WOMAC, PPT	No
9	Jorge Velázquez Saornil (2022)	RCT, Spain	60	60 ± 10/ 62 ± 10	Knee osteoarthritis	NR	DN vs. Sham DN	12 weeks	VAS, WOMAC	No
10	Juan Antonio Valera-Calero (2021)	RCT, Spain	15	24.8 ± 1.8/ 25.4 ± 2.3	Patellofemoral pain syndrome	≥ 6 months	DN + galvanic current (rectus femoris MTrP) vs. Sham DN	1 week	VAS	No
11	Mohammadreza Farazdaghi (2021)	RCT, Iran	40	61.00 ± 7.91/ 56.20 ± 6.03	Knee osteoarthritis	NR	DN (sparrow pecking technique) vs. Sham DN	2 weeks	VAS, PPT	Yes
12	Shabnam Behrangrad (2020)	RCT, Iran	54	26.4 ± 2.9/ 26.3 ± 2.7	Patellofemoral pain syndrome	>6 weeks	DN (fast-in fast-out technique) vs. Sham DN	12 weeks	NPRS, Kujala score, PPT	Yes
13	Sophie Vervullens (2021)	RCT, Belgium	61	63 ± 10/ 66 ± 10	Knee osteoarthritis	NR	DN (active + latent MTrPs) vs. Sham DN	3 days	VAS, KOOS, PPT	Yes
14	Thomas G. Sutlive (2018)	RCT, USA	60	31.1 ± 5.1/ 30.3 ± 5.5	Patellofemoral pain syndrome	NR	DN (quadriceps MTrPs) vs. Sham DN	1 week	NPRS, Kujala score	Yes
15	Xuewei Wang (2021)	RCT, China	60	64.2 ± 12.8/ 61.9 ± 11.9	Knee osteoarthritis	40.0 ± 44.3 months	CSI + electrical DN vs. CSI	12 weeks	NRS score, WOMAC_Pain, WOMAC_Functional, WOMAC Stiffness	No
16	Yan-Tao Ma (2020)	RCT, China	48	22.48 ± 2.40/ 25.14 ± 6.02	Patellofemoral pain syndrome	NR	DN (quadriceps active MTrPs) vs. Sham DN	12 weeks	VAS, Kujala score	Yes
17	Yan-Tao Ma (2023)	RCT, China	77	74.61 ± 6.43/ 75.39 ± 5.77	Knee osteoarthritis	>1 weeks	DN (active MTrPs) + ischemic compression + stretching vs. Control	6 weeks	NPRS, WOMAC_Pain, WOMAC_Functional, PPT	Yes
18	Xi Jingqi (2024)	RCT, China	74	62.8 ± 9.1/ 60.9 ± 12.5	Knee osteoarthritis	>1 months	ESWT + DN vs. ESWT	8 weeks	VAS, WOMAC, PPT	Yes
19	Johnson C. Y. Pang (2022)	RCT, China	90	60.56 ± 5.93/ 61.23 ± 5.49	Knee osteoarthritis	57.17 ± 57.09 months	US-guided DN + Exercise vs. Sham DN + Exercise	8 weeks	VAS, KOOS-Pain, KOOS-Symptoms, KOOS-QOL	Yes
20	Jorge Velázquez-Saornil (2022)	RCT, Spain	60	60 ± 10/ 62 ± 10	Knee osteoarthritis	NR	DN vs. Sham DN	12 weeks	VAS, WOMAC	No

Abbreviations: ACL, anterior cruciate ligament; CSI, corticosteroid injection; DN, dry needling; ESWT, extracorporeal shock wave therapy; GM, gluteus medius; KOA, knee osteoarthritis; KOOS, Knee Injury and Osteoarthritis Outcome Score; KSS, Knee Society Score; MTrP(s), myofascial trigger point(s); NPRS, Numeric Pain Rating Scale; NR, not reported; PFPS, patellofemoral pain syndrome; PPT, pressure pain threshold; PPTGM, pressure pain threshold of the gastrocnemius muscle; PPTQL, pressure pain threshold of the quadriceps lateralis; QOL, quality of life; RCT, randomized controlled trial; Rh, rehabilitation protocol; US, ultrasound-guided; VAS, visual analog scale; WOMAC, Western Ontario and McMaster Universities Osteoarthritis Index.

### Risk of bias assessment

**Fig 2:**
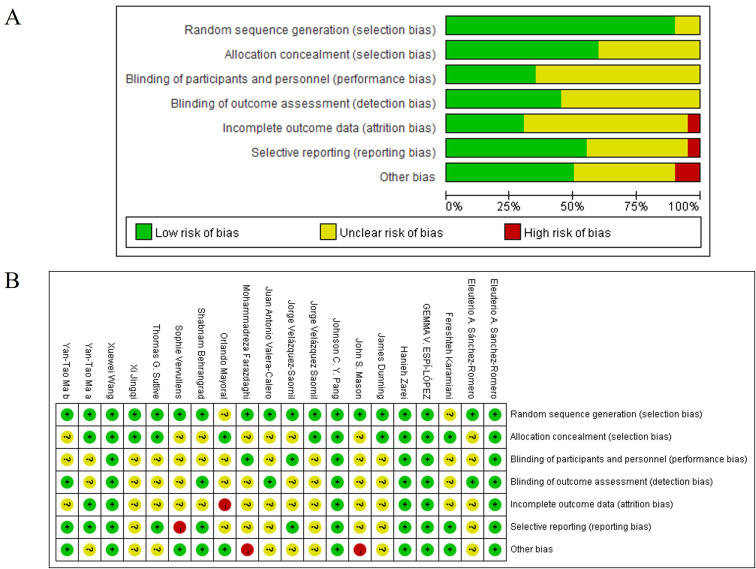
The risk of bias summary for studies included in the meta-analysis. Original figure created by author; licensed under CC BY 4.0.

Fig 2 presents the risk of bias summary. Most studies demonstrated low risk of bias for randomization processes and missing outcome data (Fig 2A). However, several studies raised “some concerns” or “high risk” in the domains of blinding of outcome assessment and selective reporting (Fig 2B). Detailed domain-level results are provided in S3 Table. Notably, concerns regarding assessor blinding were particularly relevant for subjective outcomes (e.g., VAS, NPRS, WOMAC), which may be susceptible to detection bias. The GRADE assessment (S5 Table) rated the certainty of evidence as moderate for all key outcomes, reflecting some risk of bias and heterogeneity.

### The influence of dry needling on knee pain and function

**Fig 3:**
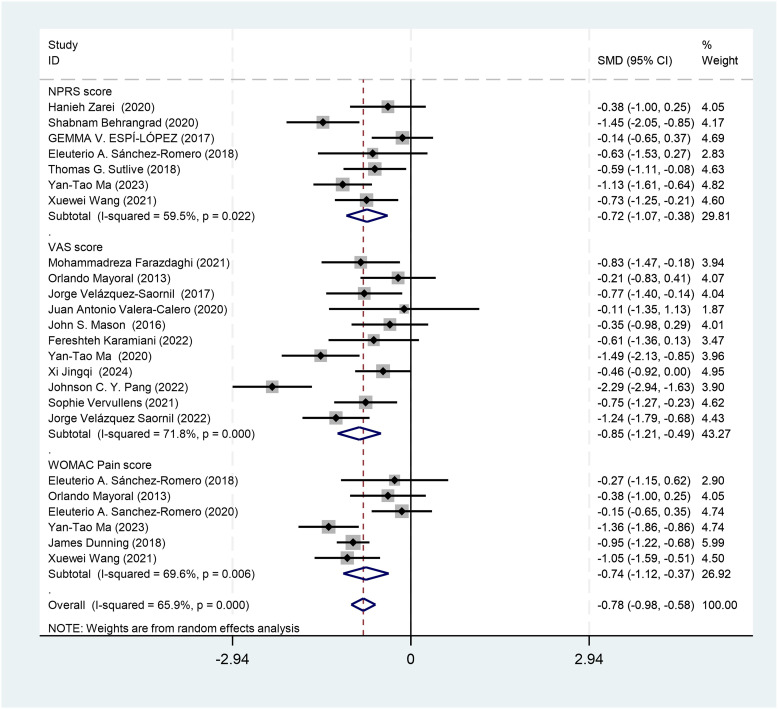
Forest plot for knee pain outcomes. Original figure created by author; licensed under CC BY 4.0.

Fig 3 displays the forest plot for the effect of DN on knee pain, stratified by pain measure (NPRS, VAS, and WOMAC Pain), using WMDs. DN significantly reduced pain intensity across all measures compared with controls: NPRS: WMD = –1.00 (95% CI: –1.25 to –0.76; *I²* = 0.0%; k = 7 studies); VAS: WMD = –1.19 (95% CI: –1.73 to –0.66; *I²* = 80.4%; k = 11 studies); WOMAC Pain: WMD = –1.76 (95% CI: –2.57 to –0.95; *I²* = 67.6%; k = 6 studies); Overall pooled: WMD = –1.25 (95% CI: –1.58 to –0.92; *I²* = 74.7%).

The direction of effect consistently favored DN across all subgroups. NPRS demonstrated no heterogeneity (*I²* = 0.0%), whereas substantial heterogeneity was observed for VAS (*I²* = 80.4%) and WOMAC Pain (*I²* = 67.6%), likely reflecting differences in patient populations, DN protocols, and follow-up durations. NPRS and VAS pain reductions of 1.00 and 1.19 points, respectively, approached the commonly accepted minimal clinically important difference (MCID) of 1.0–2.0 points on a 0–10 scale.

**Fig 4:**
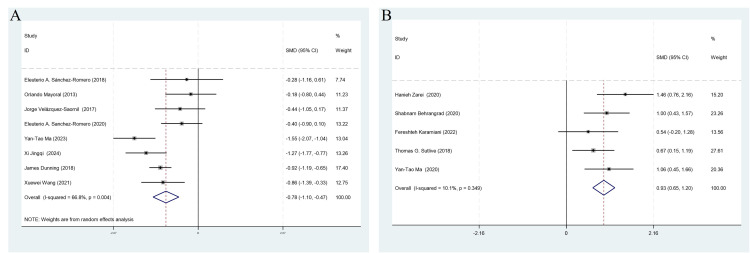
The influence of DN at myofascial trigger points in the treatment of Knee Function. **(A)** WOMAC Functional score; **(B)** Kujala score. Original figure created by author; licensed under CC BY 4.0.

Fig 4 illustrates the effect of DN on functional outcomes using WMDs. WOMAC Functional Score (Fig 4A): DN significantly improved knee function compared with controls (WMD = –6.59, 95% CI: –8.88 to –4.29; *I²* = 61.6%; k = 8 studies). This improvement falls at the lower bound of commonly cited MCID estimates (6–12 points on a 0–100 scale). Kujala Patellofemoral Score (Fig 4B): DN significantly improved function in PFPS patients (WMD = 6.39, 95% CI: 4.64 to 8.14; *I²* = 30.1%; k = 5 studies). The low heterogeneity indicates consistent results across included trials. However, this improvement falls below the commonly cited MCID threshold of approximately 8–10 points.

### Sensitivity analyses

Pre-specified sensitivity analyses using standardized mean differences (SMDs, Hedges’ g) confirmed the robustness of the primary findings (S8 Table). All SMD estimates indicated moderate to large effect sizes. The consistency between WMD and SMD results confirms that findings were not sensitive to the choice of effect measure.

### Publication bias

Funnel plots for pain outcomes appeared relatively symmetric (S1A Fig), while those for functional outcomes showed some asymmetry, particularly for the Kujala score (S1C Fig). Egger’s regression test did not indicate statistically significant publication bias for any outcome: knee pain (*p* = 0.774), WOMAC functional score (*p* = 0.214), or Kujala score (*p* = 0.408) (S2 Table). However, the limited number of studies reporting the Kujala score (k < 10) reduces the statistical power of Egger’s test for this outcome. Leave-one-out sensitivity analyses confirmed that pooled estimates remained stable following the sequential removal of individual studies (S2 Fig).

## Discussion

This meta-analysis of 20 RCTs (n = 1,234) demonstrates that DN targeting MTrPs significantly reduces knee pain and improves functional outcomes in patients with KOA and PFPS. Pain reductions were observed across all measures, with NPRS (WMD = –1.00) and VAS (WMD = –1.19) reductions approaching or exceeding commonly cited MCIDs of 1.0–2.0 points on a 0–10 scale. Functional improvements were statistically significant for both the WOMAC functional subscale (WMD = –6.59) and the Kujala score (WMD = 6.39) [38].

### Clinical relevance and implications for practice

Although all pooled estimates achieved statistical significance, clinical relevance varied across outcomes. The WOMAC functional improvement (WMD = –6.59 on a 0–100 scale) falls at the lower bound of established MCID ranges (approximately 6–12 points), indicating that the average treatment effect may be of marginal clinical significance for some patients. The Kujala score improvement (WMD = 6.39) did not consistently reach the commonly cited MCID threshold of approximately 8–10 points. Therefore, while DN produces detectable functional gains, these may not uniformly translate into clinically perceptible improvements for all patients. Pain reductions on NPRS and VAS were more encouraging, approaching or exceeding MCID thresholds in most analyses. However, substantial heterogeneity (VAS *I²* = 80.4%) and predominantly short follow-up durations limit the generalizability of these findings [39].

Collectively, the results support DN as a potential adjunctive therapy within a multimodal treatment approach for knee disorders, rather than a standalone intervention. DN may complement exercise therapy, manual therapy, and pharmacological treatments by addressing myofascial pain sources that other modalities may not directly target. However, we do not recommend routine integration of DN into clinical practice at this stage; its use should be guided by individualized clinical judgment and the presence of identifiable MTrPs.

### Mechanisms of action and biological basis

The analgesic effects of DN are thought to involve both peripheral and central mechanisms. Peripherally, needle insertion into MTrPs elicits local twitch responses, which may disrupt sustained sarcomere contraction at dysfunctional motor endplates, restore local blood flow, and reduce nociceptive biochemical mediators such as substance P and bradykinin [40,41]. Centrally, DN may modulate spinal segmental inhibition through activation of A-delta afferents, potentially attenuating central sensitization —a process implicated in both KOA and PFPS [42–44]. However, the clinical data analyzed in this review do not permit definitive conclusions regarding central neuromodulatory effects, and these mechanisms remain to be confirmed by future mechanistic studies.

### Impact of blinding limitations

A critical consideration when interpreting these findings is the difficulty of achieving adequate blinding in DN trials. The RoB 2 assessment identified concerns regarding participant and assessor blinding in the majority of included studies. Since all primary outcomes (NPRS, VAS, WOMAC, Kujala) are subjective and self-reported, they are particularly susceptible to expectancy and placebo effects. Inadequate blinding in DN trials can inflate perceived analgesic effects, raising the possibility that a proportion of the observed benefits may reflect non-specific contextual factors rather than the isolated physiological effects of needle penetration.

Several included trials employed sham DN controls, which partially mitigate this concern [45,46]. However, few trials formally assessed blinding success. Sensitivity analyses excluding studies with high risk of bias yielded slightly attenuated but still statistically significant estimates, suggesting that blinding limitations may have inflated effect sizes but are unlikely to fully account for the observed benefits. Future trials should employ validated sham procedures with documented sensory credibility, formally assess blinding success, and incorporate objective functional measures (e.g., timed up-and-go test, gait analysis) alongside subjective outcomes.

### Limitations of the current study

The findings should be interpreted in light of several limitations. First, substantial clinical and methodological heterogeneity was observed across the included studies. Variation in DN protocols (needle depth, stimulation technique, session frequency), muscles targeted, concurrent co-interventions, and participant characteristics (disease type, severity, chronicity) likely contributed to the observed between-study variability. Although pre-specified subgroup analyses by disease type and follow-up duration partially explained the observed heterogeneity, the limited number of studies within each subgroup precluded robust meta-regression.

Second, the included studies did not consistently differentiate between active and latent MTrPs. Since these may respond differently to DN, this inconsistency limits the clinical interpretability of pooled results. Future studies should explicitly classify and report the target MTrP type (active vs. latent) to improve clinical interpretability of pooled results.

Third, most trials assessed outcomes within a short timeframe (≤12 weeks), precluding conclusions regarding the durability of treatment effects. Long-term RCTs with follow-up periods of at least six months are needed.

Fourth, adverse events were inconsistently reported across studies, preventing a systematic evaluation of the safety profile of DN for knee disorders. Future research should systematically document adverse events to inform risk-benefit assessments.

Fifth, none of the included studies directly compared DN with guideline-recommended first-line treatments such as structured exercise therapy or intra-articular corticosteroid injections. Without such comparative trials, it is difficult to position DN within the treatment hierarchy or determine its relative cost-effectiveness.

Finally, the lack of standardization in DN protocols across studies limits clinical applicability. Future trials should prioritize protocol transparency and directly compare different needling techniques, stimulation modes, and treatment schedules to identify optimal parameters.

### Future research directions

Several gaps in the current evidence base should be addressed by future research. First, large-scale, adequately powered RCTs with diverse patient populations are needed to strengthen the generalizability of findings. Most included trials had small sample sizes, limiting statistical power and precision of effect estimates.

Second, long-term follow-up is critically lacking. Most trials assessed outcomes within 12 weeks, precluding conclusions about the durability of treatment effects or potential for symptom recurrence. Future studies should incorporate follow-up periods of at least six months and assess whether DN influences the progression of knee disorders, particularly KOA.

Third, head-to-head comparative trials are needed to position DN within the existing treatment hierarchy. None of the included studies directly compared DN with guideline-recommended first-line treatments such as structured exercise therapy, manual therapy, or intra-articular corticosteroid injections. Such trials should evaluate relative efficacy, durability of effects, safety, and cost-effectiveness.

Fourth, standardization of DN protocols is essential. The substantial procedural variability observed across studies—in needle depth, stimulation technique, session frequency, and treatment duration—likely contributed to the heterogeneity in treatment effects. Future trials should directly compare different needling parameters to identify optimal protocols for specific knee conditions.

Fifth, future studies should explicitly differentiate between active and latent MTrPs and investigate whether treatment responses differ by trigger point type. Additionally, systematic documentation of adverse events is needed to establish a comprehensive safety profile.

Finally, mechanistic research using objective outcome measures (e.g., electromyography, pressure algometry, gait analysis) could help clarify the peripheral and central analgesic pathways through which DN exerts its effects, enabling more targeted and evidence-based clinical application.

### Interpretation in light of clinical heterogeneity

Despite overall statistically significant findings, the meta-analysis revealed substantial between-study heterogeneity for several outcomes (*I²* often exceeding 60%), indicating that true treatment effects varied meaningfully across trials. Pre-specified subgroup analyses by disease type (KOA vs. PFPS) and follow-up duration partially explained some variability, but considerable heterogeneity persisted. Potential sources include: (1) variation in DN protocols (needle depth, stimulation mode, session frequency); (2) differences in muscles targeted; (3) concomitant interventions; (4) participant characteristics (diagnosis, severity, age, activity level); and (5) differences in outcome measurement timing and instruments.

Given this heterogeneity, caution is warranted when extrapolating pooled estimates to individual patients or clinical settings. Future trials should standardize DN protocols and outcome measures and, where feasible, conduct individual patient data meta-analyses or meta-regression to identify specific moderators of treatment response. Our conclusions should be understood as reflecting potential short-term benefits within a multimodal approach, with the magnitude and durability of clinically meaningful effects likely varying across patients and contexts.

## Conclusion

This meta-analysis of 20 RCTs suggests that DN targeting MTrPs provides significant short-term pain relief and functional improvement in patients with KOA and PFPS, with pain reductions approaching clinically important thresholds. However, substantial heterogeneity, blinding limitations, and predominantly short follow-up durations necessitate cautious interpretation. DN should be considered as a potential adjunctive therapy guided by individualized clinical judgment rather than a routine standalone treatment. High-quality, adequately powered RCTs with standardized protocols, rigorous blinding, and extended follow-up are needed to confirm sustained efficacy and establish the role of DN within the treatment hierarchy for knee disorders.

## Supporting information

S1 File Complete literature search strategy. (DOCX)

S2 File PRISMA 2020 Checklist. (DOCX)

S1 Table Key features and design of the included trials. (DOCX)

S2 Table Regression analysis results for knee pain bias and function outcomes. (DOCX)

S3 Table Cochrane RoB 2 (Risk of Bias 2) assessment for the included randomized controlled trials (RCTs). (DOCX)

S4 Table Heterogeneity statistics (Tau-squared) and model selection for meta-analyses. (DOCX)

S5 Table GRADE analysis: Overall quality assessment and summary of findings comparing Dry Needling to Sham Dry Needling for knee osteoarthritis/patellofemoral pain. (DOCX)

S6 Table List of excluded studies and the reasons for their exclusion from the systematic review. (DOCX)

S7 Table Results of subgroup meta-analyses for disease type and follow-up duration on knee pain and functional outcomes. (DOCX)

S8 Table A summary table presenting the baseline characteristics (demographic and clinical) of the participants in the included randomized controlled trials. It includes data on sample size, age, sex distribution, diagnosis, duration of symptoms, and key clinical outcome scores at baseline.
(DOCX)

S1 Fig Publication bias funnel plots. (JPG)

S2 Fig Sensitivity analyses. (JPG)
