# Supplementary material for: The effectiveness of dry needling at myofascial trigger points for knee disorders: A quantitative synthesis of randomized controlled trials
Source: PLoS One. 2026 Apr 10;21(4):e0346129. doi: 10.1371/journal.pone.0346129 (PMC13068212; doi:10.1371/journal.pone.0346129)
Supplement: S1 File — (DOCX) [file pone.0346129.s001.docx]

PubMed

#1 "Dry Needling"[Mesh] OR "dry needling"[tiab] OR "intramuscular stimulation"[tiab]

#2 "Myofascial Trigger Point"[Mesh] OR "myofascial trigger point"[tiab] OR "trigger point"[tiab]

#3 "Knee Joint"[Mesh] OR "Knee Injuries"[Mesh] OR "Osteoarthritis, Knee"[Mesh] OR "Patellofemoral Pain Syndrome"[Mesh]

OR "knee pain"[tiab] OR "knee disorder"[tiab] OR "knee osteoarthritis"[tiab] OR "KOA"[tiab] OR "patellofemoral pain"[tiab] OR "PFPS"[tiab]

#4 randomized controlled trial[pt] OR controlled clinical trial[pt] OR randomized[tiab] OR randomised[tiab] OR randomly[tiab]

OR placebo[tiab] OR trial[tiab]

#5 #1 AND #2 AND #3 AND #4

Embase

#1 'dry needling'/exp OR 'dry needling':ti,ab OR 'intramuscular stimulation':ti,ab

#2 'myofascial trigger point'/exp OR 'myofascial trigger point':ti,ab OR 'trigger point':ti,ab

#3 'knee joint'/exp OR 'knee injury'/exp OR 'knee osteoarthritis'/exp OR 'patellofemoral pain syndrome'/exp

OR 'knee pain':ti,ab OR 'knee disorder':ti,ab OR 'PFPS':ti,ab OR 'KOA':ti,ab

#4 'randomized controlled trial'/exp OR 'controlled clinical trial'/exp OR randomized:ti,ab OR randomised:ti,ab OR randomly:ti,ab OR placebo:ti,ab OR trial:ti,ab

#5 #1 AND #2 AND #3 AND #4

Cochrane Library

#1 MeSH descriptor: [Dry Needling] explode all trees OR "dry needling":ti,ab,kw OR "intramuscular stimulation":ti,ab,kw

#2 MeSH descriptor: [Myofascial Trigger Point] explode all trees OR "myofascial trigger point":ti,ab,kw OR "trigger point":ti,ab,kw

#3 MeSH descriptor: [Knee Joint] explode all trees OR MeSH descriptor: [Knee Injuries] explode all trees OR MeSH descriptor: [Osteoarthritis, Knee] explode all trees OR MeSH descriptor: [Patellofemoral Pain Syndrome] explode all trees

OR "knee pain":ti,ab,kw OR "knee osteoarthritis":ti,ab,kw OR "patellofemoral pain":ti,ab,kw OR "PFPS":ti,ab,kw OR "KOA":ti,ab,kw

#4 "randomized controlled trial":pt OR randomized:ti,ab,kw OR randomised:ti,ab,kw OR randomly:ti,ab,kw OR trial:ti,ab,kw OR placebo:ti,ab,kw

#5 #1 AND #2 AND #3 AND #4

Web of Science (Core Collection)

TS=("dry needling" OR "intramuscular stimulation")

AND TS=("myofascial trigger point" OR "trigger point")

AND TS=("knee pain" OR "knee disorder" OR "knee osteoarthritis" OR "KOA" OR "patellofemoral pain" OR "PFPS")

AND TS=(randomized OR randomised OR "controlled trial" OR placebo OR trial)

China National Knowledge Infrastructure (CNKI)

主题 = (“干针” OR “肌内刺激”)

AND 主题 = (“肌筋膜触发点” OR “触发点”)

AND 主题 = (“膝关节疼痛” OR “膝关节疾病” OR “膝骨关节炎” OR “髌股疼痛综合征”)

AND 主题 = (“随机对照试验” OR “临床试验”)
