# Supplementary material for: The effectiveness of dry needling at myofascial trigger points for knee disorders: A quantitative synthesis of randomized controlled trials
Source: PLoS One. 2026 Apr 10;21(4):e0346129. doi: 10.1371/journal.pone.0346129 (PMC13068212; doi:10.1371/journal.pone.0346129)
Supplement: S1 Table — (DOCX) [file pone.0346129.s003.docx]

| **No.** | **Author (Year)** | **Study Design, Region** | **Sample Size (T/C)** | **Age (T/C)** | **Enrolled Patient** | **Pain Duration** | **Intervention (Obs. Group)** | **Intervention (Ctrl. Group)** | **Follow-Up** | **Outcome Index** |
| --- | --- | --- | --- | --- | --- | --- | --- | --- | --- | --- |
| 1 | Shabnam Behrangrad (2020) | RCT, Iran | 54 | 26.4±2.9 / 26.3±2.7 | PFPS | >6 weeks | DN using fast-in/fast-out + vertical motion | Ischemic compression | 12 weeks | NPRS, Kujala, PPT |
| 2 | James Dunning (2018) | RCT, Italy | 242 | 57.1±13.2 / 58.1±13.1 | KOA | 4.5±4.7 yrs | MT + exercise + electrical DN | MT + exercise only | 12 weeks | WOMAC Pain, Function, Stiffness |
| 3 | GEMMA V. ESPÍ-LÓPEZ (2017) | RCT, USA | 60 | 29.2±10.5 / 29.7±9.5 | PFPS | ≥6 months | MT + exercise + DN to quadriceps | MT + exercise only | 12 weeks | NPRS, KSS function |
| 4 | Mohammadreza Farazdaghi (2021) | RCT, Iran | 40 | 61.0±7.91 / 56.2±6.03 | KOA | NR | DN with sparrow pecking/coning | Sham DN (plastic mimic) | 2 weeks | VAS, PPT |
| 5 | Xi Jingqi (2024) | RCT, China | 74 | 62.8±9.1 / 60.9±12.5 | KOA | >1 month | Shockwave + DN | Shockwave or DN alone | 8 weeks | VAS, WOMAC, PPT |
| 6 | Fereshteh Karamiani (2022) | RCT, Iran | 29 | 24.4±3.94 / 27.64±7.13 | PFPS | >4 weeks | Physio + TrP-DN | Physio only | 1 week | VAS, Kujala |
| 7 | Yan-Tao Ma (2020) | RCT, China | 48 | 22.48±2.40 / 25.14±6.02 | PFPS | NR | DN to quadriceps TrPs | Sham DN (non-penetrating) | 12 weeks | VAS, Kujala |
| 8 | Yan-Tao Ma (2023) | RCT, China | 77 | 74.61±6.43 / 75.39±5.77 | KOA | >1 week | DN + ischemic compression + stretching | No intervention specified | 6 weeks | NPRS, WOMAC Pain, Function, PPT |
| 9 | John S. Mason (2016) | RCT, USA | 39 | 20.3±1.08 / 20.16±2.12 | Atraumatic knee pain | >2 weeks | DN to hamstring TrPs | Sham DN (non-penetrating) | 1 week | VAS, PPT |
| 10 | Orlando Mayoral (2013) | RCT, Spain | 40 | 71.65±6.06 / 72.9±7.85 | Myofascial pain syndrome | NR | True DN to MTrPs | Sham DN | 24 weeks | VAS, WOMAC Pain, Function, PPT |
| 11 | Johnson C. Y. Pang (2022) | RCT, China | 90 | 60.56±5.93 / 61.23±5.49 / 61.97±5.39 | KOA | 57.17±57.09 months | US-guided DN + exercise + education | Exercise + education only | 8 weeks | VAS, KOOS Pain, Symptoms, QOL |
| 12 | Eleuterio A. Sánchez-Romero (2018) | RCT, Spain | 20 | 71.89±4.80 / 70.89±3.21 | KOA | >30 days | Exercise + DN | Exercise + Sham DN | 12 weeks | NPRS, WOMAC Pain, Function |
| 13 | Eleuterio A. Sanchez-Romero (2020) | RCT, Spain | 62 | 72.97±6.29 / 71.65±5.00 | KOA | NR | Exercise + DN to LE MTrPs | Exercise + Sham DN | 48 weeks | WOMAC Pain, Function, Stiffness |
| 14 | Thomas G. Sutlive (2018) | RCT, USA | 60 | 31.1±5.1 / 30.3±5.5 | PFPS | NR | DN to quadriceps using standardized protocol | Sham DN (no penetration) | 1 week | NPRS, Kujala |
| 15 | Juan Antonio Valera-Calero (2021) | RCT, Spain | 15 | 24.8±1.8 / 25.4±2.3 | PFPS | ≥6 months | DN + galvanic current (HIPE/LIPE) | Sham DN (non-penetrating tube) | 1 week | VAS |
| 16 | Jorge Velázquez-Saornil (2017) | RCT, Spain | 42 | 31.4±8.3 / 34.4±8.6 | Post-ACL surgery | NR | Rehab + DN to vastus medialis TrP | Rehab only | 5 weeks | VAS, WOMAC, PPT |
| 17 | Jorge Velázquez Saornil (2022) | RCT, Spain | 60 | 60±10 / 62±10 | KOA | NR | DN | HA injection or ultrasound + isometric | 12 weeks | VAS, WOMAC |
| 18 | Sophie Vervullens (2021) | RCT, Belgium | 61 | 63±10 / 66±10 | KOA | NR | DN to active + latent MTrPs | Sham DN (subcutaneous only) | 3 days | VAS, KOOS, PPT |
| 19 | Xuewei Wang (2021) | RCT, China | 60 | 64.2±12.8 / 61.9±11.9 | KOA | 40.0±44.3 months | CSI + electrical DN | CSI + exercise | 12 weeks | NRS, WOMAC Pain, Function, Stiffness |
| 20 | Hanieh Zarei (2020) | RCT, Iran | 40 | 22.25±3.25 / 25.65±8.49 | PFPS | NR | Exercise + DN | Exercise only | 6 weeks | NPRS, Kujala, PPTQL, PPTGM |
