# Supplementary material for: The effectiveness of dry needling at myofascial trigger points for knee disorders: A quantitative synthesis of randomized controlled trials
Source: PLoS One. 2026 Apr 10;21(4):e0346129. doi: 10.1371/journal.pone.0346129 (PMC13068212; doi:10.1371/journal.pone.0346129)
Supplement: S2 Table — (DOCX) [file pone.0346129.s004.docx]

Supplementary Table 2

| Std_Eff | Coefficient | Std. err. | t | P>\|t\| | [95% conf. interval] |
| --- | --- | --- | --- | --- | --- |
| Knee pain bias  Knee Function  WOMAC Functional score  Kujala score | -.2865921  4.356085  1.581732 | .9836466  3.137622  1.647737 | -0.29  1.39  0.96 | 0.774  0.214  0.408 | -2.32655 1.753366  -3.3214 12.03357  -3.662104 6.825568 |
