# Supplementary material for: The effectiveness of dry needling at myofascial trigger points for knee disorders: A quantitative synthesis of randomized controlled trials
Source: PLoS One. 2026 Apr 10;21(4):e0346129. doi: 10.1371/journal.pone.0346129 (PMC13068212; doi:10.1371/journal.pone.0346129)
Supplement: S3 Table — (DOCX) [file pone.0346129.s005.docx]

Supplementary Table 3

| Cochrane RoB 2 | Bias arising from the randomization process | Bias due to deviations from intended interventions  (effect of assignment to intervention) | Bias due to deviations from intended interventions  (effect of adhering to intervention) | Bias due to missing outcome data | Bias in measurement of the outcome | Bias in selection of the reported result |
| --- | --- | --- | --- | --- | --- | --- |
| Hanieh Zarei (2020)  Shabnam Behrangrad (2020)  Mohammadreza Farazdaghi (2021)  GEMMA V. ESPÍ-LÓPEZ (2017)  Eleuterio A. Sánchez-Romero (2018)  Orlando Mayoral (2013)  Jorge Velázquez-Saornil (2017)  Eleuterio A. Sanchez-Romero (2020)  Juan Antonio Valera-Calero (2021)  John S. Mason (2016)  Fereshteh Karamiani (2022)  Thomas G. Sutlive (2018)  Yan-Tao Ma (2020)  Yan-Tao Ma (2023)  Xi Jingqi (2024)  James Dunning (2018)  Xuewei Wang (2021)  Johnson C. Y. Pang (2022)  Sophie Vervullens (2021)  Jorge Velázquez Saornil (2022) | Low 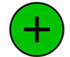  Low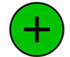  Low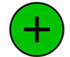  Low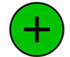  Low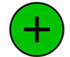  Low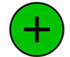  Low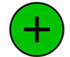  Low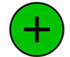  Low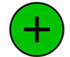  Low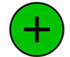  Some concerns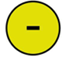  Low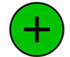  Low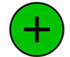  Low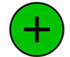  Low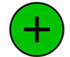  Low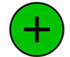  Low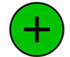  Low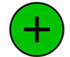  Low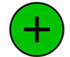  Low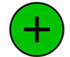 | Low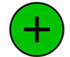  Low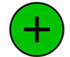  Some concerns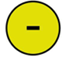  Low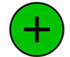  Low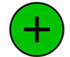  Some concerns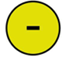  Low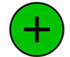  Some concerns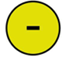  Some concerns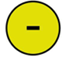  Some concerns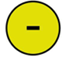  Low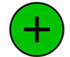  Low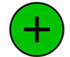  Low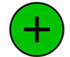  Some concerns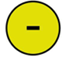  Low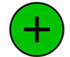  Low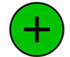  Low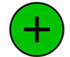  Low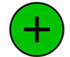  Low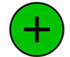  Some concerns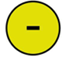 | Low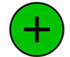  Some concerns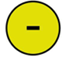  Some concerns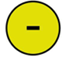  Low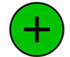  Low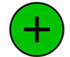  Low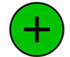  Some concerns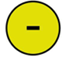  Some concerns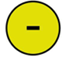  Low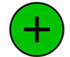  Some concerns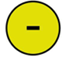  Some concerns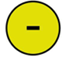  Some concerns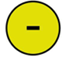  Some concerns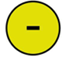  Some concerns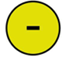  Some concerns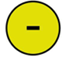  Some concerns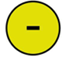  Low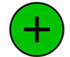  Low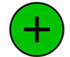  Some concerns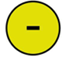  Low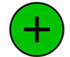 | Low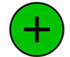  Low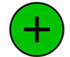  Some concerns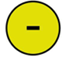  Low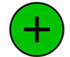  Low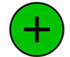  High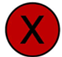  Some concerns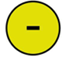  Some concerns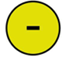  Some concerns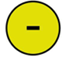  Some concerns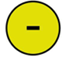  Some concerns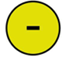  Some concerns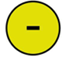  Some concerns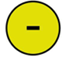  Low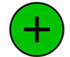  Some concerns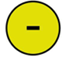  Some concerns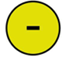  Low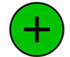  Low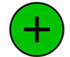  Some concerns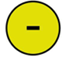  Some concerns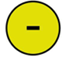 | Low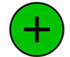  Low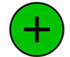  Low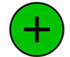  Low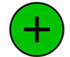  Low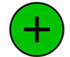  Some concerns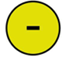  Some concerns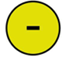  Low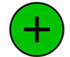  Some concerns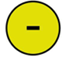  Some concerns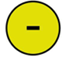  Low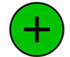  Low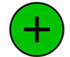  Some concerns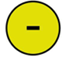  Some concerns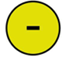  Some concerns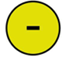  Some concerns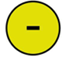  Low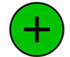  Low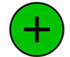  High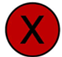  Low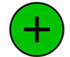 | Low  Some concerns  High  Low  Low  Some concerns  Some concerns  Some concerns  Some concerns  High  Low  Some concerns  Some concerns  Low  Some concerns  Some concerns  Low  Low  Some concerns  Some concerns |
