# Supplementary material for: The effectiveness of dry needling at myofascial trigger points for knee disorders: A quantitative synthesis of randomized controlled trials
Source: PLoS One. 2026 Apr 10;21(4):e0346129. doi: 10.1371/journal.pone.0346129 (PMC13068212; doi:10.1371/journal.pone.0346129)
Supplement: S4 Table — (DOCX) [file pone.0346129.s006.docx]

Supplementary Table 4

| Std_Eff | τ² (Tau-squared) | Model Type |
| --- | --- | --- |
| Knee pain bias  Knee Function  WOMAC Functional score  Kujala score | 0.2219  0.1763  0.0000 | Random-effects  Random-effects  Fixed-effects |
