# Supplementary material for: The effectiveness of dry needling at myofascial trigger points for knee disorders: A quantitative synthesis of randomized controlled trials
Source: PLoS One. 2026 Apr 10;21(4):e0346129. doi: 10.1371/journal.pone.0346129 (PMC13068212; doi:10.1371/journal.pone.0346129)
Supplement: S5 Table — (DOCX) [file pone.0346129.s007.docx]

| **Supplementary table 5. GRADE analysis: overall quality assessment and summary of findings** | | | | | |
| --- | --- | --- | --- | --- | --- |
| **Dry needling compared to Sham dry needling for knee osteoarthritis** | | | | | |
| **Patient or population**: Patients with knee osteoarthritis or patellofemoral pain syndrome  **Intervention**: Dry needling at myofascial trigger points  **Comparison**: Sham dry needling or standard physical therapy | | | | | |
| Outcomes | **Anticipated absolute effects^*^** (95% CI) | Relative effect (95% CI) | № of participants  (studies) | Certainty of the evidence (GRADE) | Comments |
| Pain reduction (NPRS/VAS) | Moderate to large reduction in pain | **WMD −1.25 (−1.58 to −0.92)** | 696 cases  672 controls (20 RCTs) | ⨁⨁◯◯ MODERATE | Some risk of bias and heterogeneity observed; however, consistency across studies supports clinical relevance. |
| Functional improvement (WOMAC) | Improvement in functional scores | **WMD −6.59 (−8.88 to −4.29)** | 310 cases  300 controls (8 RCTs) | ⨁⨁◯◯ MODERATE | High heterogeneity and some unclear risk of bias, but positive and consistent effect across trials. |
| Functional improvement (Kujala) | Large improvement in patellofemoral function | **WMD 6.39 (4.64 to 8.14)** | 117 cases  114 controls (5 RCTs) | ⨁⨁◯◯ MODERATE ^a^ | Asymmetry in funnel plot suggests possible publication bias; robust sensitivity analysis supports the findings. |
| ***The risk in the intervention group** (and its 95% confidence interval) is based on the assumed risk in the comparison group and the **relative effect** of the intervention (and its 95% CI).  **CI:** Confidence interval; WMD: Weighted mean difference | | | | | |
| **GRADE Working Group grades of evidence** **High certainty:** We are very confident that the true effect lies close to that of the estimate of the effect **Moderate certainty:** We are moderately confident in the effect estimate: The true effect is likely to be close to the estimate of the effect, but there is a possibility that it is substantially different **Low certainty:** Our confidence in the effect estimate is limited: The true effect may be substantially different from the estimate of the effect **Very low certainty:** We have very little confidence in the effect estimate: The true effect is likely to be substantially different from the estimate of effect | | | | | |
