# Supplementary material for: The effectiveness of dry needling at myofascial trigger points for knee disorders: A quantitative synthesis of randomized controlled trials
Source: PLoS One. 2026 Apr 10;21(4):e0346129. doi: 10.1371/journal.pone.0346129 (PMC13068212; doi:10.1371/journal.pone.0346129)
Supplement: S6 Table — (DOCX) [file pone.0346129.s008.docx]

Supplementary Table 6. Excluded Studies and Reasons for Exclusion

| No. | First Author (Year) | Title of Study | Reason for Exclusion | Notes | Reference ID |
| --- | --- | --- | --- | --- | --- |
| 1  2  3  4  5 | Dunning,J (2019)  Lin xinzhen (2022)  Luo Xu (2024)  Zhang Jiahao (2023)  Li Liwen (2023) | Dry needle therapy is used to treat knee osteoarthritis  The influence of acupuncture inactivating myofascial pain trigger points on the infrared thermal imaging characteristics and clinical efficacy of KOA patients  Clinical research progress of Acupuncture Treatment for Knee osteoarthritis  Research on the Central Regulatory Mechanism of Classical Acupuncture and Dry Acupuncture in the Treatment of Chronic Pain in Knee OsteoArthritis Based on fMRI-ALFF  Research Progress on Dry Needle Therapy for Knee Osteoarthritis | No valid data  Non-randomized controlled trial  Conference abstract  Review article  Review article | Insufficient statistical outcome reporting  Did not meet inclusion criteria (RCT only)  No full-text available for data extraction  Secondary literature  Did not report original data | - Dunning,J, He Chengqi. Dry Needle Therapy for Knee Osteoarthritis [J]. Chinese Rehabilitation,2019,34(03):126. - Lin Xingzhen, Zhu Manhua, Peng Tianzhong, et al. The influence of acupuncture inactivating myofascial pain trigger points on the infrared thermal imaging characteristics and clinical efficacy of KOA patients [J]. Jilin Medical Journal,2022,43(07):1750-1753. - Luo Xu, Pei Jiuguo Research progress of acupuncture in the treatment of knee osteoarthritis [C] / / the Chinese acupuncture society. 2024 Chinese acupuncture and moxibustion societies annual meeting proceedings.2024:1681-1684. The DOI: 10.26914 / Arthur c. nkihy. 2024.039530. - Zhang Jiahao. Research on the Central Regulatory Mechanism of Classical Acupuncture and Dry Acupuncture in the Treatment of Chronic Pain in Knee OsteoArthritis Based on fMRI-ALFF [D] Henan university of Chinese medicine, 2023. DOI: 10.27119 /, dc nki. Ghezc. 2023.000207. - Li Liwen, Zheng Hui, Hua Can, et al. The research progress of dry needle treatment of knee osteoarthritis [J]. Journal of practical internal medicine of traditional Chinese medicine, 2023, 5 (11) : 61-63. The DOI: 10.13729 / j.i SSN. 1671-7813. Z20221791. |
