# Supplementary material for: The effectiveness of dry needling at myofascial trigger points for knee disorders: A quantitative synthesis of randomized controlled trials
Source: PLoS One. 2026 Apr 10;21(4):e0346129. doi: 10.1371/journal.pone.0346129 (PMC13068212; doi:10.1371/journal.pone.0346129)
Supplement: S7 Table — (DOCX) [file pone.0346129.s009.docx]

Supplementary Table S6.

Subgroup Meta-Analyses for Disease type and Follow-up Duration

| Subgroup Study | WMD (95% CI) | I-squared |
| --- | --- | --- |
| Knee pain (Disease type)  NPRS score (PFPS)  NPRS score (KOA)  VAS score (PFPS)  VAS score (KOA)  VAS score (MPS)  WOMAC Pain score (KOA)  WOMAC Pain score (MPS)  Knee pain (follow-up time)  NPRS score (> 4 weeks)  NPRS score (> 4 weeks)  VAS score (< 4 weeks)  VAS score (> 4 weeks)  WOMAC Pain score (> 4 weeks)  Knee WOMAC Functional score (Disease type)  PFPS  KOA  MPS  Knee WOMAC Functional score (follow-up time)  > 4 weeks  Knee Kujala score (Disease type)  PFPS  KOA  Knee Kujala score (follow-up time)  > 4 weeks  < 4 weeks | **-1.25(-1.58,-0.92)**  -0.8 (-1.16,-0.57)  -1.35(-1.82,-0.89)  -1.17(-2.39,0.06)  -1.30(-1.99,-0.62)  -0.40(-1.58,0.78)  -1.84(-2.72,-0.96)  -1.09(-2.87,0.69)  **-1.25(-1.58,-0.92)**  -0.70(-1.30,-0.10)  -1.07(-1.34,-0.79)  -0.97(-1.49,-0.45)  -1.35(-2.14,-0.57)  -1.76(-2.57,-0.96)  **-6.59(-8.88,-4.29)**  -9.43(-12.21,-6.65)  -6.52(-9.20,-3.84)  -1.73(-7.72,4.26)  **-6.59(-8.88,-4.29)**  -6.59(-8.88,-4.29)  **6.39 (4.64,8.14)**  5.96(4.11,7.81)  9.99(4.65,15.33)  **6.39 (4.64,8.14)**  6.57(4.60,8.53)  5.72(1.87,9.58) | **74.7**  0.0  0.0  78.1  85.9  0.0  71.8  0.0  **74.7**  0.0  0.0  8.8  89.1  67.6  **61.6**  0.0  60.8  0.0  **61.6**  61.6  **30.1**  20.3  0.0  **30.1**  62.5  0.0 |

Abbreviations:MPS: *Myofascial Pain Syndrome*; KOA: *Knee Osteoarthritis*; PFPS: *Patellofemoral Pain Syndrome*.
