# Supplementary material for: The effectiveness of dry needling at myofascial trigger points for knee disorders: A quantitative synthesis of randomized controlled trials
Source: PLoS One. 2026 Apr 10;21(4):e0346129. doi: 10.1371/journal.pone.0346129 (PMC13068212; doi:10.1371/journal.pone.0346129)
Supplement: S8 Table — It includes data on sample size, age, sex distribution, diagnosis, duration of symptoms, and key clinical outcome scores at baseline. (DOCX) [file pone.0346129.s010.docx]

**Fig 1. The influence of dry needling at myofascial trigger points in the treatment of Knee pain.**


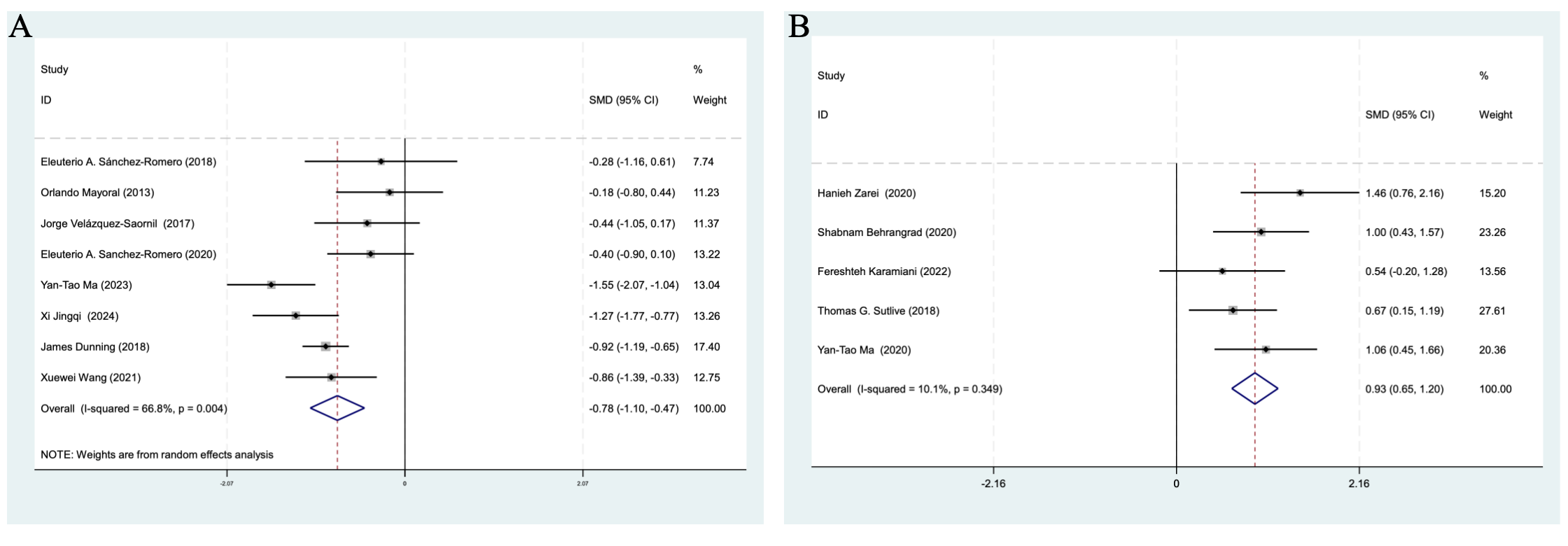


**Fig 2. The influence of dry needling at myofascial trigger points in the treatment of Knee Function. (A) WOMAC Functional score; (B) Kujala score.**
