# Supplementary figures and images for: The effectiveness of dry needling at myofascial trigger points for knee disorders: A quantitative synthesis of randomized controlled trials
Source: PLoS One. 2026 Apr 10;21(4):e0346129. doi: 10.1371/journal.pone.0346129 (PMC13068212; doi:10.1371/journal.pone.0346129)

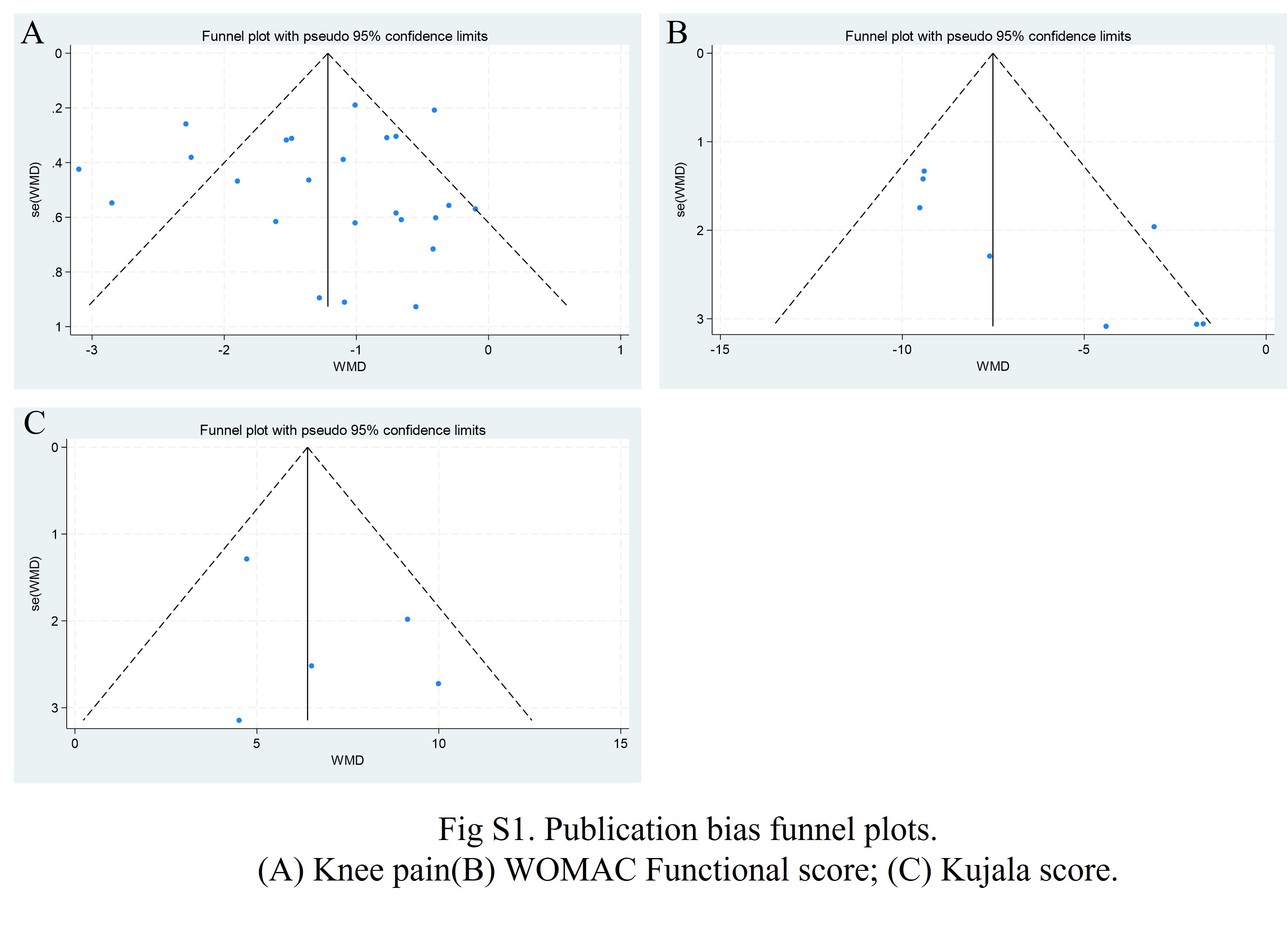

Supplement: S1 Fig — (JPG) [file pone.0346129.s011.jpg]

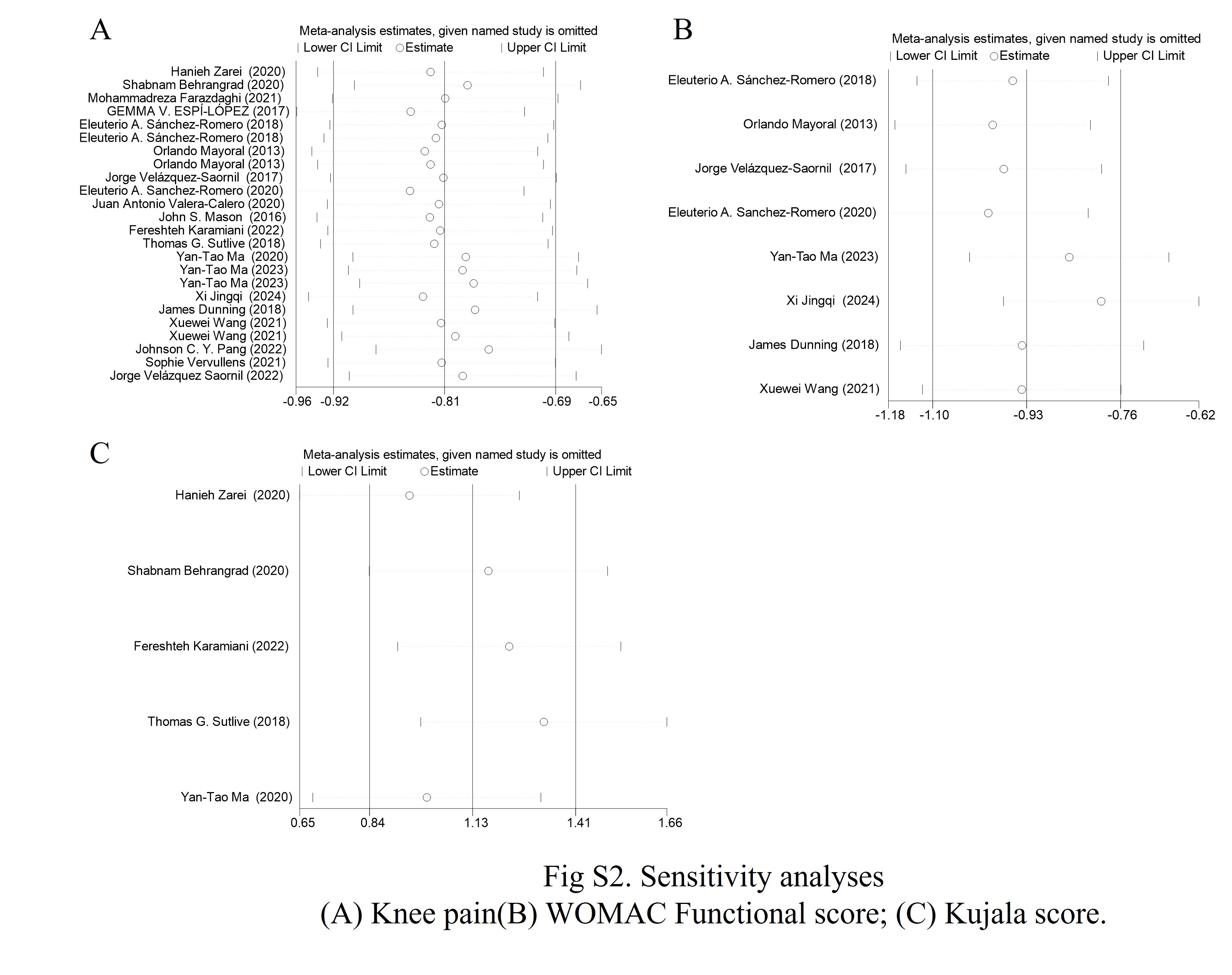

Supplement: S2 Fig — (JPG) [file pone.0346129.s012.jpg]
